# Integration of full divertor detachment with improved core confinement for tokamak fusion plasmas

**DOI:** 10.1038/s41467-021-21645-y

**Published:** 2021-03-01

**Authors:** L. Wang, H. Q. Wang, S. Ding, A. M. Garofalo, X. Z. Gong, D. Eldon, H. Y. Guo, A. W. Leonard, A. W. Hyatt, J. P. Qian, D. B. Weisberg, J. McClenaghan, M. E. Fenstermacher, C. J. Lasnier, J. G. Watkins, M. W. Shafer, G. S. Xu, J. Huang, Q. L. Ren, R. J. Buttery, D. A. Humphreys, D. M. Thomas, B. Zhang, J. B. Liu

**Affiliations:** 1 Institute of Plasma Physics, Chinese Academy of Sciences, Hefei, China; 2 General Atomics, San Diego, CA USA; 3 Oak Ridge Associated Universities, Oak Ridge, TN USA; 4 Lawrence Livermore National Laboratory, Livermore, CA USA; 5 Sandia National Laboratories, Livermore, CA USA; 6 Oak Ridge National Laboratory, Oak Ridge, TN USA

**Keywords:** Nuclear fusion and fission, Magnetically confined plasmas, Techniques and instrumentation

## Abstract

Divertor detachment offers a promising solution to the challenge of plasma-wall interactions for steady-state operation of fusion reactors. Here, we demonstrate the excellent compatibility of actively controlled full divertor detachment with a high-performance (*β*_N_ ~ 3, *H*_98_ ~ 1.5) core plasma, using high-β_p_ (poloidal beta, *β*_p_ > 2) scenario characterized by a sustained core internal transport barrier (ITB) and a modest edge transport barrier (ETB) in DIII-D tokamak. The high-*β*_p_ high-confinement scenario facilitates divertor detachment which, in turn, promotes the development of an even stronger ITB at large radius with a weaker ETB. This self-organized synergy between ITB and ETB, leads to a net gain in energy confinement, in contrast to the net confinement loss caused by divertor detachment in standard H-modes. These results show the potential of integrating excellent core plasma performance with an efficient divertor solution, an essential step towards steady-state operation of reactor-grade plasmas.

## Abstract (web summary)

Plasma fusion devices like tokamaks are important for energy generation but there are many challenges for their steady state operation. Here, the authors show that full divertor detachment is compatible with high-confinement high-poloidal-beta core plasmas and this prevents the damage to the divertor target plates and the first wall.

## Introduction

One of the key challenges facing the economic operation of fusion reactors is to sustain a high-temperature high-pressure plasma with sufficient confinement time while preventing damage to the plasma-facing components, including the divertor plates and first wall. The excessively high heat flux on the divertor plates must be actively handled. To meet the critical requirement for long-pulse operation of the ITER^1–5^, divertor detachment is proposed as the most promising solution for steady-state plasma–wall interactions^6^. In existing tokamaks, divertor detachment is routinely obtained by either injecting fuel particles or impurities to enhance the divertor power dissipation and thus reduce plasma temperature at the divertor plates^6^. When the plasma temperature falls below a few eV, the enhanced atomic processes move the plasma boundary interaction off the divertor target, which is the signature of divertor detachment. In addition, fusion plasmas in future tokamak reactors require both a hot core for fusion reaction and self-sustained noninductive current for steady-state operation. The formation of a transport barrier in the plasma not only elevates performance in the core region but also increases the noninductive bootstrap current, both of which reduce the requirement of external heating and current drive and thus improve the fusion economy^7–10^. In this respect, ITER will adopt the high confinement (H-mode) plasmas with a spontaneous edge transport barrier (ETB) as the baseline scenario to achieve its scientific goal of steady-state operation^11–13^. However, in most present tokamaks, it is commonly found that divertor detachment significantly reduces the plasma confinement, as the detachment front cools the core plasma through degrading the H-mode ETB (or pedestal)^14–20^. The compatibility of divertor detachment and noninductive high-confinement advanced scenarios requires urgent investigation. Most of the present investigations focus on improving the core-edge-divertor integration by mitigating the pedestal reduction resulting from the divertor detachment.

In this paper, we show that in the high-*β*_p_ scenario plasmas, the normally degraded pedestal due to divertor detachment does not degrade the global performance and instead, facilitates the achievement of a strong internal transport barrier (ITB) at a large radius. Hence, we have achieved fully detached divertor plasmas simultaneously with a sustained high-confinement core at normalized performance approaching reactor-relevant levels in the DIII-D facility^21^, as manifested by *H*_98_ ~ 1.5, *β*_N_ ~ 3, *β*_P_ > 2, and *β*_T_ ~ 2–2.5%. Here, *H*_98_ = *τ*_exp_/*τ*_scaling_ is the energy confinement enhanced factor, $\beta _{\mathrm{T}} = \frac{p}{{B_{\mathrm{T}}^2/2\mu _0}}$ is the toroidal beta, $\beta _{\mathrm{p}} = \frac{p}{{B_{\mathrm{p}}^2/2\mu _0}}$ is the poloidal beta, and $\beta _{\mathrm{N}} = \frac{p}{{B^2/2\mu _0}}\frac{{aB_{\mathrm{T}}}}{{I_{\mathrm{p}}}}$ is the normalized beta, where *B* is the total magnetic field, *B*_T_ is the toroidal magnetic field, *B*_p_ is the poloidal magnetic field, *I*_p_ is the plasma current, *a* is the minor radius, *p* is the plasma pressure, *τ*_exp_ is the experimental energy confinement time and *τ*_scaling_ is the ITPA scaling for the H-mode energy confinement time^22^. These divertor plasmas are well detached, with low plasma temperature *T*_e_ ≤ 5 eV across the entire divertor target and low steady-state divertor particle and heat fluxes.

## Results

### Detached high-*β*_p_ plasmas with N_2_ seeding

**Fig. 1:**
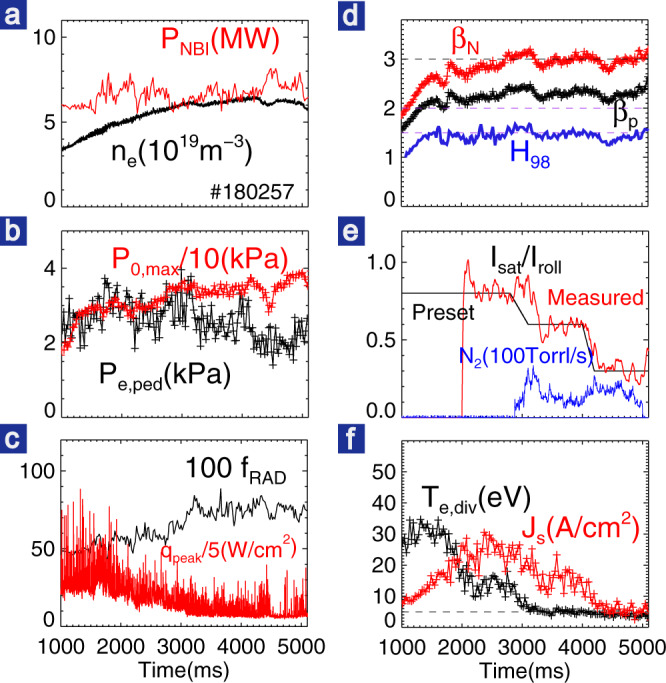
Plasma parameters for a high-βp discharge (#180257) with active feedback control of detachment via N_2_ seeding. **a** NBI heating power (red) and line-averaged density. **b** The peak electron pressure (red) and pedestal top electron pressure (black) measured by the Thomson scattering system. **c** Fraction of radiation/NBI power and IR peak heat flux (red). **d** *β*_N_ (red), *β*_p_ (black), and *H*_98_ (blue). **e** Preset and measured *I*_sat_/*I*_roll_ for divertor detachment feedback control, nitrogen gas puffing rate. **f** Peak particle flux and *T*_e_ near the outer strike point, with 1 A/cm^2^ corresponding to particle flux of 6.24 × 10^22^ m^−2^ s^−1^.

Figure 1 shows an example of detached high-*β*_p_ plasmas under active control via impurity seeding in DIII-D. In this discharge with *I*_p_ ~ 0.72MA, a biased-up quasi-double-null shape with the radial distance between upper divertor separatrix and lower divertor separatrix at the outer midplane *dR*_sep_ ~ 8 mm (>2*λ*_q_) and outer strike point on the upper ceiling divertor are utilized. Several plasma feedback control systems were used in order to achieve stationary high confinement. D_2_ gas injection is adjusted to feedback control the pedestal top density in order to avoid excessive gas fueling. In this discharge, the line-averaged density is ~90% of the Greenwald density limit (*n*_GW_ = *I*_p_/*πa*^2^). When the energy confinement varies during the discharge, for example, because of ITB formation, a constant heating power could lead to a rapid *β*_N_ increase, challenging the magnetohydrodynamic (MHD) stability limits. To avoid the beta collapse due to MHD limits, a preset *β*_N_ target waveform is feedback controlled by adjusting the beam power automatically. During the plasma current flattop, with ~7–8 MW neutral beam injection heating, we have achieved *β*_N_ ~ 3, *β*_p_ > 2, and *H*_98_ ~ 1.5 which are close to the requirements of previous ITER steady-state scenarios^23^, although with a plasma edge safety factor (pitch of the magnetic field lines) *q*_95_ ~ 7–8 which is higher but close to the target value of *q*_95_ ~ 6.7 found in recent state-of-the-art modeling of ITER’s steady-state scenario based on the high poloidal beta approach^24^. Here, *q*_95_ corresponds to the safety factor in the edge where the normalized poloidal flux is 95%. The noninductive current, mainly comprised of the bootstrap current in both ETB (or pedestal) and ITB plus a small fraction of beam current drive, constitutes more than 70% of total plasma current, with the ohmic current fraction <30% and the loop voltage *V*_loop_ < 100 mV during the plasma current flattop. Low or even zero ohmic currents is ultimately desired for long-pulse operation. The high-*β*_p_ scenario has lower disruption risk due to higher *q*_95_ and significant advantages for driving the bootstrap current, which is highly desired for steady-state operation. Extensive efforts have been made in DIII-D to develop high-*β*_p_ scenario plasmas^25–31^.

Real-time active control of divertor detachment has been achieved by impurity seeding feedback optimization, in addition to high core plasma confinement. The controller utilizes the ion saturation current (*I*_sat_) measured by divertor Langmuir probes around the outer strike point. The degree of detachment (DoD)^32^ is calculated in real-time by comparing the outer target peak *I*_sat_ measurement to the maximum *I*_sat_ value at its rollover, i.e., *I*_roll_. The feedback allows control of the detachment gradually with avoidance of excessive gas puffing which may cause strong confinement degradation. In this discharge, nitrogen (N_2_) impurity seeding is performed and *I*_roll_ is the maximum *I*_sat_ value before impurity seeding. The experimentally measured *I*_sat_/*I*_roll_ or 1/DoD closely follows the preset waveform shown in Fig. 1e, demonstrating the success of the active detachment control system.

**Fig. 2:**
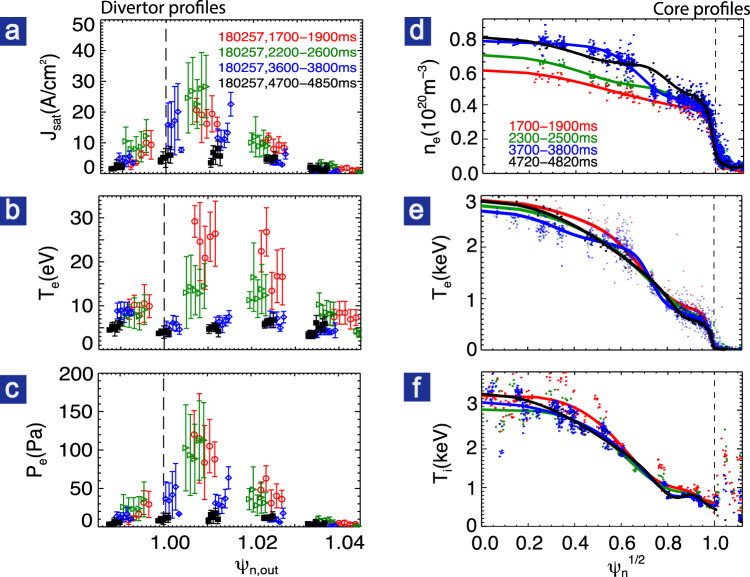
Divertor profiles (left) and core plasma profiles (right) for attached divertor (red), high-recycling divertor (green), pronounced detachment (blue) and full detachment (black). **a**–**c** Divertor particle flux, electron temperature, and electron pressure with statistical error bars also shown. **d**–**f** Core electron density, electron temperature, and ion temperature. Electron density and temperature are measured by the Thomson Scattering system. Ion temperature is measured by charge–exchange recombination system for C6+ impurity ions. The EFIT strike point in (**a**–**c**) was shifted by 0.5% *ψ*_n_ to match probes.

As shown in Fig. 1e, we achieved stable divertor detachment with DoD ~1.5 during 3.2–4 s and DoD ~3.3 during 4–5 s. After the N_2_ injection, the steady-state peak heat flux measured from an infrared camera (Fig. 1c), as confirmed by the divertor Langmuir probe, is reduced by >85% and reached about 0.3 MW/m^2^, very close to the measurement threshold. The reduction of the heat flux is due to the reduction of both particle flux and plasma temperature. As can be seen in Fig. 2b, after the onset of detachment, the peak electron temperature *T*_e_ measured from outer divertor Langmuir probes embedded in the target plates is reduced from 25 to <5 eV across the entire divertor target plate. In addition, the peak particle flux (Fig. 2a) $J_{{\mathrm{sat}}} = I_{{\mathrm{sat}}}/S_{{\mathrm{probe}}} = en_{\mathrm{e}}C_{\mathrm{s}} = en_{\mathrm{e}}\sqrt {\left( {T_{\mathrm{e}} + T_{\mathrm{i}}} \right)/m_{\mathrm{i}}}$ is reduced from ~25 to 15 A/cm^2^ when DoD ~ 1.5 and further to ~5 A/cm^2^ when DoD > 3. The inner divertor also exhibits cold and detached even before the N_2_ puffing, which is mainly due to the strong in-out divertor asymmetry driven by the *E* × *B* drift flow^6,33^. The lower divertor target is far away from the main separatrix, and thus has much lower (by an order of magnitude) particle and heat fluxes and lower temperature (*T*_e_ < 10 eV) even before the N_2_ seeding.

Note that *J*_sat_ both near the strike point and in the far scrape-off layer (SOL) is significantly reduced, indicating “full detachment” across the target plate. The electron pressure (Fig. 2c) calculated based on Langmuir probe measurements exhibits about 90% loss near the strike point, compared to that before impurity seeding, suggesting the strong pressure detachment. Note that *T*_e_ < 5 eV is highly desirable for suppression of erosion in reactor-grade devices.

**Fig. 3:**
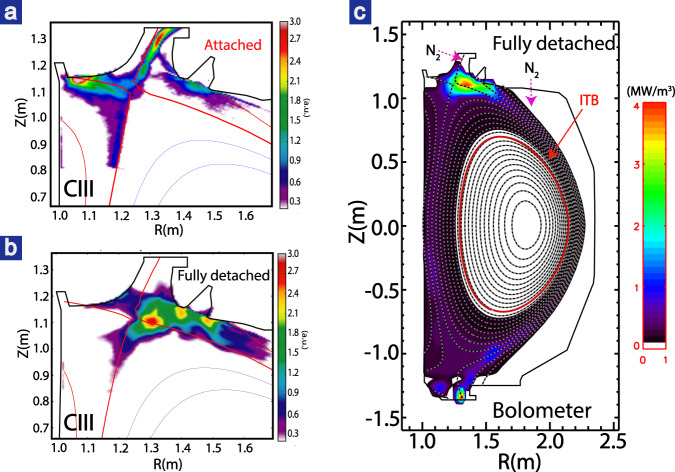
2D radiation measured by bolometer (right) and CIII radiation from Tangential TV (left). Here, a.u. means arbitrary units. Both show the radiation peaks near the X-point during full detachment (**b**, **c**) with respect to the attachment (**a**) whose radiation peaks almost at the divertor plates. Note that the reconstructed bolometer radiation near the lower divertor target has a large uncertainty since several lower bolometer chords are cut-off by the shelf tiles. N_2_ puffing locations are shown as the purple arrows.

The seeded impurity enhances the radiation that dissipates the power towards the divertor and thus eventually provides access to divertor detachment. As measured by the bolometer, ~80% of the injected heating power was dissipated by the radiation during full detachment (Fig. 1c). The full detachment here has been further confirmed by the CIII radiation (Fig. 3a, b), which moves away from the divertor target plates and peaking near the X-point forming an “X-point MARFE”^20,34–38^. The two-dimensional (2D) radiation inferred by the bolometer also exhibits the significant peak radiation around X-point, as shown in Fig. 3c. The neutral pressure in the divertor volume increases greatly after the onset of detachment and further increases when moving towards deeper detachment, which is beneficial for pumping and thus particle and impurity control.

The high global confinement in the high-*β*_p_ plasmas is mainly due to the simultaneously sustained ITB and ETB, with the former one dominant. As can be seen in Fig. 2e, f, both the ion and electron temperature profiles in the core plasma are almost identical whether the divertor is attached or detached. A strong ITB with a peak gradient around *ρ* ~ 0.6–0.7 can be observed in both *T*_e_ and *T*_i_ profiles. No clear ITB is observed in the density radial profile when the divertor attaches, while with impurity injection, a weak density ITB forms and moves outward when full divertor detachment is achieved. The core profiles indicate that the improvement in particle confinement is weaker than the energy confinement, which benefits impurity exhaust and prevents impurity concentration^39–41^. In addition, a closed divertor with baffled geometry preventing recycling neutrals escaping from divertor region is beneficial for impurity screening^42^. As a result, *Z*_eff_ is about 3 in this discharge and core radiation remains low (25% of total heating power) even during full detachment. It should be noted that similar plasmas with similar properties for both global confinement and detachment but lower *Z*_eff_ (<2.4) have been achieved by using impurity seeding from divertor volume. In addition, as shown in Fig. 3, the radiation peak is localized near the X-point and is still far away from the ITB foot. The low radiation in the core plasma is beneficial for the achievement of high confinement.

### Advantages of the high-*β*_p_ scenario for improving core-edge integration

**Fig. 4:**
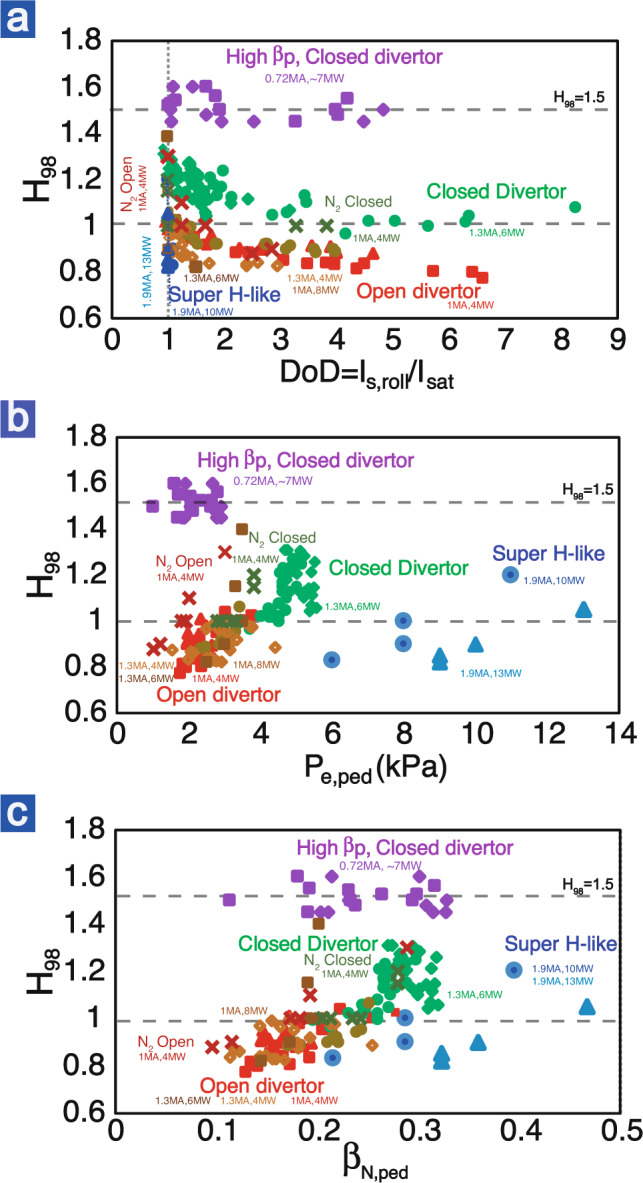
The global energy confinement quality under different detached conditions. The *H*_98_ versus degree of detachment (**a**), pedestal pressure (**b**), and pedestal *β*_N,ped_ (c) across several plasma scenarios. The scenarios compared are open divertor standard H-mode (red), closed divertor standard H-mode (green), super H-like (blue), and high-*β*_p_ (purple) plasmas. The power and plasma current are also labeled. *β*_N,ped_ = *β*_ped_/(*I*_p_/*aB*_T_) takes different plasma current and *B*_T_ into account for a better comparison of the pedestal pressure. The plasma shapes for open and closed geometries can be found in refs. ^43,45^. The purple-diamond data in high-*β*_p_ are taken from Fig. 1 and purple-square data are from a similar discharge.

In standard H-mode plasmas without an ITB, i.e., having an ETB only, the divertor detachment normally leads to a significant reduction of the pedestal and the global confinement, as shown in Fig. 4a. The global confinement degradation is mainly attributed to the pedestal reduction (Fig. 4b, c), since the core pressure profile is stiff and thus the global confinement is linearly proportional to the pedestal height. This pedestal degradation due to divertor detachment is difficult to avoid in conventional H-mode discharges in present medium-size tokamaks. High divertor density during divertor detachment leads to high pedestal density and collisionality which degrades the pedestal pressure due to the reduction of bootstrap current^43,44^. In addition, the high density in the detached divertor reduces the neutral ionization mean-free-path at the pedestal, shifting the pedestal pressure gradient outwardly towards the higher *q* region and leading to a lower pedestal pressure MHD stability limit.

Divertor closure facilitates detachment with a higher pedestal pressure but not enough to maintain the core confinement in standard H-mode. With an open divertor, in DIII-D standard H-mode, the detachment window compatible with a high confinement core is very narrow, with *H*_98_ dropping from above 1 to below 0.9 when DoD > 2. In very high pedestal plasmas (Super H like^45^), the detachment window almost disappears (blue data in Fig. 4a). A closed divertor facilitates the achievement of divertor detachment at a lower pedestal density^46,47^ and thus higher pedestal pressure^43^, as illustrated in Fig. 4b, c. Thus, the *H*_98_ > 1 can be still obtained until DoD > 4. Note that even with a closed divertor, the *H*_98_ in standard H-mode decreases with DoD as well. More excitingly, the high-*β*_p_ plasma exhibits much better compatibility between divertor detachment and a high-confinement core. As can be seen in Fig. 4a, in the high-*β*_p_ discharges, much better plasma confinement (*H*_98_ ~ 1.5) can be stably maintained even with DoD ~ 5–6. This is mainly attributed to the high-*β*_p_ scenario breaking the correlation between the pedestal and global confinement. The decoupling of core and pedestal via ITB allows high confinement compatible with divertor detachment.

### The synergy between ITB and ETB

**Fig. 5:**
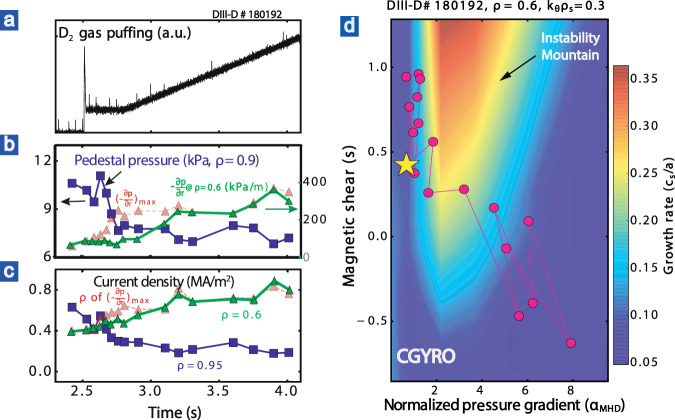
A detached high-*β*_p_ discharge with D_2_ gas puffing only. **a** D_2_ puffing rate. **b** Pedestal pressure, peak core pressure gradient (red), and pressure gradient at *ρ* = 0.6 (green). **c** Plasma current density at the pedestal, at peak gradient region (red), and at *ρ* = 0.6 (green). **d** The growth rate of instability calculated from Gyrokinetic simulation code CGYRO^65^, as scanned by the normalized pressure gradient (*α*_MHD_ ∝ *Rq*^2^∂p/∂r) and magnetic shear (*s* = *r*∂q/∂r/*q*). The experimental points are shown as the red dots, the experimental equilibrium (*t* ~ 2.75 s) used for simulation is marked as the yellow star, and the weak ITB cases are at the left-top side while the strong ITB cases are at the right-bottom side. *k*_*θ*_*ρ*_*s*_ = 0.3 was selected for simulation since experimental fluctuation measurements identify similar fluctuations with similar wavelengths. Note that the outer divertor is marginally detached at 2.7 s < *t* < 3.5 s and pronouncedly detached at *t* > 3.5 s.

**Fig. 6:**
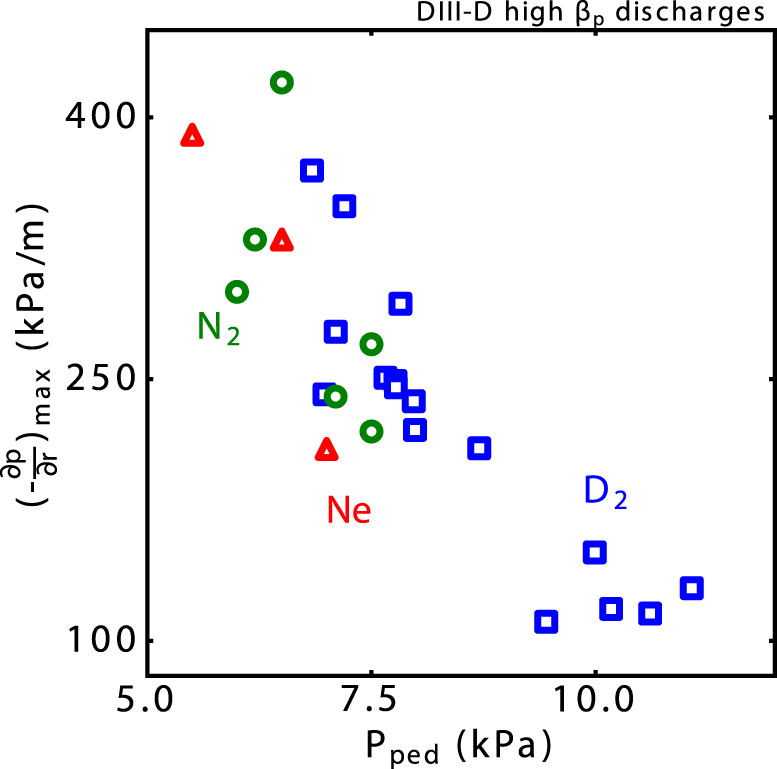
The peak pressure gradient at the ITB region versus the pedestal pressure, showing that a strong ITB is associated with a weak pedestal. Nitrogen (green circle), neon (red triangle), and D_2_ (blue) are shown.

Furthermore, instead of causing core confinement loss, the divertor detachment-induced degradation of pedestal pressure in the high-*β*_p_ scenario facilitates the achievement of a strong ITB at a large radius and thus promotes high global confinement. Figure 5a–c illustrates a dedicated high-*β*_p_ discharge for natural detachment access with density ramping up using D_2_ gas puffing only. As shown in Figs. 5b and 6, a weak pedestal is associated with a strong ITB, while a high pedestal is associated with a weak or no ITB in these high-*β*_p_ plasmas. This is similar to the results discussed in ref. ^48^, where an edge-localized mode (ELM) crash is observed to lower the pedestal and trigger a transition from a high pedestal to no ITB state, into a self-organized state with a strong ITB and a low pedestal, thus leading to higher confinement intermittently. However, here in this study, the pedestal reduction leading to the transition into a strong ITB state is mainly due to the divertor detachment access.

It has been shown that the two states, i.e., the strong-ITB dominated state with a low pedestal and the high-pedestal dominated state with a weak ITB, are controlled by the interaction of the bootstrap current (and its effect on the magnetic shear), and the kinetic ballooning mode (KBM) instability boundary^48,49^. With continuously increased D_2_ gas puffing, the pedestal pressure (Fig. 5b) is decreased, and, in turn, drives a strong ITB with high core pressure gradient at the large radius (*ρ* ∼ 0.6) via a self-organized feedback loop. The reduced pedestal decreases the edge current density (Fig. 5c). Given the constant total plasma current, the current density at a large radius is increased so that the magnetic shear is decreased. As confirmed by the gyrokinetic simulation (Fig. 5d and Supplementary Figs. 1 and 2 in Supplementary Note), with continuously reduced magnetic shear *s* (*s* = *r*∂q/∂r/*q*), the plasma equilibrium evolves from low to high *α* (*α* ∝ −*Rq*^2^∂p/∂r) at *ρ* ~ 0.6, by bypassing the area of high instability growth rate in the *s*–*α* parameter space (“instability mountain”). The enhanced Shafranov shift due to the high *β*_p_ is beneficial for stabilizing the drift-wave-like turbulence^50^. When the magnetic shear becomes weak enough that KBM starts to enter the second stable region, the pressure gradient increases to form a core ITB. The increased gradient generates bootstrap current and further weakens the magnetic shear at the ITB region. This positive feedback loop benefits the formation and growth of a strong ITB. The strong ITB and simultaneously weak ETB are strongly self-organized. The other state with a high pedestal and a weak ITB is also self-organized^49^. The strong ETB lowers the shear in the pedestal region, increases the shear at the top of ETB, and forms another positive feedback loop between the KBM and edge bootstrap current in the pedestal region. The divertor detachment lowers the pedestal pressure and thus prevents the positive feedback loop from forming a high pedestal and a weak ITB. Instead, it facilitates the achievement and sustainment of a strong ITB at a large radius (*ρ* ∼ 0.6), which improves the confinement. Note that such correlation between the pedestal and the ITB has been observed in various high-*β*_p_ divertor detachment experiments, either detached plasmas by using D_2_ alone or with N_2_ seeding, or with Neon seeding, as shown in Fig. 6.

It is worth pointing out that another scenario with a strong ITB (not at large radius) and low confinement (L-mode, no ETB) edge has only been achieved at a relatively low effective fusion yield and *β*_N_^51^. An L-mode edge with strong turbulence leads to significant energy transport and much lower confinement. The existence of an ETB even at a low height can upshift the core density and pressure profiles and thereby improve the global performance and the energy confinement to *H*_98_ > 1.

Another advantage of the high-ITB-dominated self-organized state is that the weak ETB is more likely to operate in a small-ELM regime with much less intermittent heat flux deposited on the divertor plates, as shown in Fig. 1c. Such high-frequency benign ELMs are beneficial for the particle exhaust without causing serious impurity concentration and plasma–wall interaction issues. In contrast, a strong ETB is usually associated with low-toroidal-mode-number giant ELMs, which not only causes excessively high heat flux and serious PWI issues but also results in relaxation oscillations over a wide radial range of the plasma.

More importantly, the high-confinement high-*β*_p_ scenario with simultaneous weak ETB and strong ITB is also advantageous for achieving divertor detachment. The improved confinement with ITB reduces the heating power requirement for sustained high performance and thus decreases both the threshold of radiative impurity amount and separatrix density, which is beneficial for the achievement of divertor detachment. The required separatrix density at detachment onset strongly correlates with the plasma current and heating power^52,53^. In addition, a high *q*_95_ associated with the high-*β*_p_ scenario increases the magnetic connection length to dissipate the power via radiation at a large volume. The reduced heating power into the SOL with the economical operation, due to improved core confinement, decreases the required separatrix density at the onset of divertor detachment at a constant impurity concentration.

The N_2_ seeding further helps achieve full divertor detachment. The injected impurity increases the radiation and facilitates the achievement of detachment at a further lower upstream density^46^. In addition, based on the two-point model^6^, the downstream electron temperature at the divertor plate can be expressed as ${T}_{\mathrm{et}} \propto \frac{{\left( {1 - f_{{\mathrm{rad}}}} \right)^2}}{{\left( {1 - f_{{\mathrm{mom}},{\mathrm{loss}}}} \right)^2}}$, and divertor particle flux ${\Gamma}_{\mathrm{t}} \propto \frac{{\left( {1 - f_{{\mathrm{mom}},{\mathrm{loss}}}} \right)^2}}{{\left( {1 - f_{{\mathrm{rad}}}} \right)}} \propto \left( {1 - f_{{\mathrm{rad}}}} \right)/T_{{\mathrm{et}}}$, where *f*_rad_ is the radiation power fraction and *f*_mom,loss_ is the momentum loss factor. N_2_ seeding increases the impurity radiation and reduces the plasma temperature to below a few eV inside the divertor. This significantly enhances hydrogenic atomic processes, such as charge exchange and recombination, resulting in strong momentum loss^54^. Thus, the particle flux could be significantly reduced even at a similar *T*_et_, which facilitates the achievement of full divertor detachment without causing a highly collisional core plasma. Furthermore, it was found recently that N_2_ seeding can enhance the recombination process and thus is beneficial for the momentum dissipation and ultimately full detachment^55,56^.

**Fig. 7:**
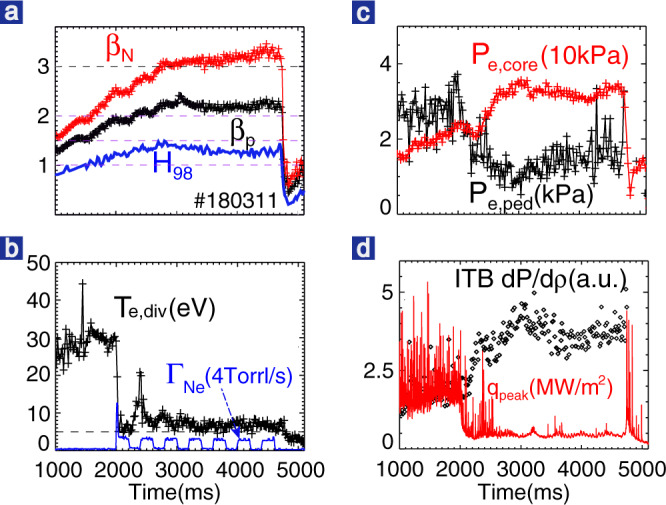
Plasma parameters for a high-*β*_p_ discharge with detachment via neon seeding. **a** *β*_N_ (red), *β*_p_ (black), and *H*_98_ (blue). **b** Neon gas puffing rate (blue) and divertor *T*_e_ near the outer strike point. **c** The peak electron pressure (red) and pedestal top electron pressure measured by the Thomson Scattering diagnostic system. **d** ITB pressure gradient and peak heat flux measured by an IR camera.

The advantages of the self-organized detached state with a strong ITB and a weak pedestal in the high-*β*_p_ plasma are further confirmed by the results of detachment experiments using neon seeding, as clearly shown in Fig. 7 for a discharge with *q*_95_ ~6.9 which is close to ITER’s steady-state scenario. Neon injection greatly enhances the radiation both in the core and in the divertor region and leads to a partially detached divertor with low heat flux (Fig. 7d) and electron temperature of <10 eV (Fig. 7b). The high radiation strongly cools the pedestal and leads to a ~70% reduction of pedestal pressure, i.e., from ~3 to ~1 kPa (Fig. 7c). Even with strong pedestal degradation, a high-confinement core is still achieved, i.e., *β*_N_ ~3 and *β*_p_ > 2 in this discharge. In addition, with neon injection, the pedestal reduction-induced ITB enhancement exhibits a more gradual behavior with a timescale of 1 s, confirming the strong correlation between pedestal reduction and ITB enhancement in Fig. 6. It should be noted here that the neon seeding successfully mitigates the ELMs and induces a long-duration ELM-less phase with only a few benign bursts (Fig. 7d). During the ELM-less period, the divertor and the pedestal maintain stationary conditions. This regime with high-confinement high-*β*_p_ core, detached divertor, and no/small ELMs may open an even better path for core-edge integration without the intermittent high heat flux from the ELMs.

## Discussion

We have achieved excellent integration of full divertor detachment with high-confinement high-*β*_p_ plasmas, maintained at *β*_N_ ~ 3, *β*_p_ > 2, and *H*_98_ ~ 1.5. The high-*β*_p_ plasmas exhibit long-sought compatibility between detachment and a high-beta high-confinement core, potentially solving one of the most challenging issues for economical fusion energy. Impurity injection, long connection length associated with high *q*_95_, closed divertor, and reduced heating power requirement thanks to high confinement, facilitate the achievement of full divertor detachment at lower edge plasma density and benefit the core-edge integration. The degraded pedestal due to divertor detachment promotes the plasma transitioning into a self-organized state with a low pedestal and a strong ITB which greatly elevates the core confinement and thus improves the core-edge integration. These results confirm the high-*β*_p_ scenario as a highly promising approach towards integration of a high-confinement core with an edge solution able to prevent damage to the divertor target plates and first wall in tokamaks. The next step is to use these results to validate self-consistent simulations of the integrated core-edge-divertor solution and extend the detached high-*β*_p_ scenario to next-step fusion experiments such as ITER.

## Methods

### DIII-D tokamak

The DIII-D tokamak is the largest magnetic fusion experiments in the United States, supported by the U.S. Department of Energy Office of Science. The tokamak consists of a toroidal vacuum chamber surrounded by coils that produce the magnetic field to confine and shape the plasma. It has a major radius of 1.67 m and a minor radius of 0.67 m, with a toroidal magnetic field of up to 2.2 T. The plasma is created by applying a voltage to ionize a small amount of gas injected into the vacuum chamber and drive a large, toroidal electrical current. The plasma is then quickly heated to a high temperature by injection of high-power neutral beams (<16 MW), while additional gas fueling increases the density. More information can be found in ref. ^57^.

### Techniques to obtain profiles

Core electron density and temperature are measured by a high-resolution Thomson Scattering system with multi-pulse lasers^58,59^. The ion temperature profiles are measured by charge-exchange recombination spectroscopy system for C6+ impurity ions at points along the outboard midplane^60^. To obtain more accurate profiles, data from the same phase of several inter-ELM periods are combined after first mapping to magnetic flux surface by using equilibria constructed from data at the time of the measurement. The equilibria used for profile mapping are produced with the EFIT code^61^ based on data from the magnetic field and poloidal flux measurements at the vessel wall.

Divertor plasma profiles are measured from divertor Langmuir probes embedded in the divertor target plates^62^. The probe tips are dome-type shape with 6 mm diameter, 1 mm height above tile surface, and ~1.5 cm separation at outer divertor target plate. They are operated as a single-probe mode with a 1 kHz sweeping frequency. A multi-point median filter was applied to the data from each probe and the error bars were given by calculating the median absolute deviation.

### Kinetic equilibria

A so-called “kinetic” equilibria, where the magnetic reconstruction is constrained by pressure profile measurements, is generated for the stability calculations. In the kinetic equilibrium, the pressure is taken from the experimental total pressure (including the measured electron and ion profiles, as well as NUBEAM^63^ modeled fast ion pressure), the core current profiles are determined from the motional Stark effect measurement, the edge current profiles are constrained by a modeled current profile and the plasma shape is determined from the magnetic field and poloidal flux measurements along the vacuum vessel walls. The edge current density profiles are the sum of the bootstrap current estimated from the Sauter expression^64^, the neutral beam is driven current, the Ohmic current which is determined from the neoclassical model, minus a small poloidal current. An iteration was carried out to recalculate the current density and readjust the pressure profiles to match the original experimental profile.

### Gyrokinetic simulation

The CGYRO^65^ is used in the gyrokinetic analysis. The linear initial value solver is employed to calculate the most unstable mode in the selected region (radial position at *ρ* = 0.6, turbulence wavelength at *k*_θ_*ρ*_s_ = 0.3). The flux-tube simulations use full gyrokinetic treatment for both electron and ion species. The calculation is based on the well-converged reconstructed equilibrium using EFIT code^61^ and the kinetic equilibria approach described above. Carbon is the major impurity considered in the modeling. The simulations in this study use the experimental profiles and equilibrium with exact shaping parametrization, electromagnetic effects *A*_||_ and *B*_||_ and collisions.

## Supplementary information

Supplementary Information

## Data Availability

Raw data were generated at the DIII-D facility. Derived data that support the plots within this paper and other findings of this study are available from the corresponding author upon reasonable request.

## Code availability

The computer code used to generate results that are reported in the paper is available from the authors on reasonable request.
