# Supplementary material for: Integration of full divertor detachment with improved core confinement for tokamak fusion plasmas
Source: Nat Commun. 2021 Mar 1;12:1365. doi: 10.1038/s41467-021-21645-y (PMC7921092; doi:10.1038/s41467-021-21645-y)
Supplement: Supplementary file 1 — Supplementary Information [file 41467_2021_21645_MOESM1_ESM.pdf]

Supplemental Info

In addition to the example shown in Fig. 5 of main text, we have also carried out simulations based on many different experimental time points, each time point updating the kinetic equilibrium reconstruction and the CGYRO calculations, and the results are similar to what is shown in Fig. 5(d) of main text. Therefore, for simplicity and readability, we only show CGYRO modeling for one representative example in the paper. Please find below two other examples. Supplementary Figure 1 shows the 2D contour based on experimental data at 2629ms, while Supplementary Figure 2 is based on the kinetic equilibrium at 2592ms. Note that the experimental data used for the gyrokinetic simulation is marked by the yellow star. The numerical values of the growth rate by CGYRO for the different cases could be different due to different plasma profiles. As can be seen, the physics picture of instability mountain and the experimental data around it is robust.

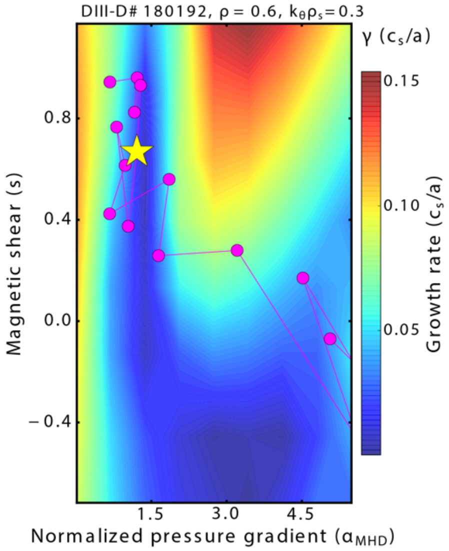

Supplementary Figure 1. s - α instability contour based on experimental data at 2629 ms.

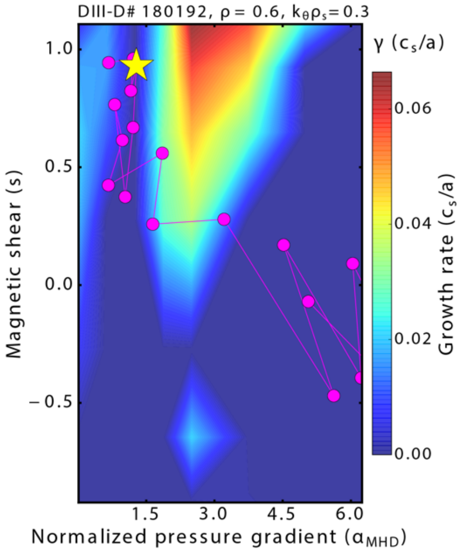

Supplementary Figure 2. s - α instability contour based on experimental data at 2592 ms. Note that we have excluded an electron MTM mode (i.e. shear <0) in this plot. The dominant mode at the region of shear >0 is KBM.
